# Waiting for Care: Length of Stay for ED Mental Health Patients by Disposition, Diagnosis, and Region (2009–2015)

**DOI:** 10.7759/cureus.25604

**Published:** 2022-06-02

**Authors:** Laura Simko, Natalia E Birgisson, Elizabeth A Pirrotta, Ewen Wang

**Affiliations:** 1 Department of Emergency Medicine, Stanford University School of Medicine, Palo Alto, USA; 2 Department of Psychiatry, Stanford University Hospital, Palo Alto, USA; 3 Department of Emergency Medicine, Stanford University School of Medicine, Stanford, USA

**Keywords:** co-occurring disorders, emergency psychiatry, length of stay, emergency mental health, mental health crisis

## Abstract

Objective

Emergency departments (EDs) face increasing mental health visits on a backdrop of insufficient mental health resources. We study ED length of stay (LOS) and disposition by 1) mental health vs. medical visits; 2) psychiatric vs. substance use visits; and 3) the four regions of the United States.

Methods

We used weighted data from the National Hospital Ambulatory Medical Care Survey (2009-2015). Visits by patients ages 18-64 were categorized into mental health and medical groups. The mental health group was then subdivided into psychiatric, substance use, and co-occurring disorders. The LOS was compared by disposition. Mental health vs. medical LOS and disposition were examined across four regions of the US.

Results

An estimated 28 million mental health and 526 million medical visits were included in the study. Mental health visits had a median (interquartile range [IQR]) of 3.7 (4.7) hours while medical visits had a median (IQR) of 2.6 (2.7) hours. Mental health compared to medical visits were more likely to result in admission or transfer and to last >6 and >12 hours. Mental health visits resulting in transfer had the longest LOS with a median (IQR) of 6.23 (7.7) hours. Of mental health visit types, co-occurring disorders visits were more likely to be >6 and >12 hours regardless of disposition. Across US regions, there was significant variation in disposition patterns for mental health vs. medical visits. The odds of mental health visits lasting >6 and >12 hours were greatest in the Northeast and the least in the South with a median (IQR) of 4.6 (5.8) hours and 3.3 (4.0) hours, respectively.

Conclusions

Metal health compared to medical visits had longer LOS, especially when the patient had co-occurring disorders or required transfer. Regionally, there is a large variation in disposition for mental health vs. medical visits. This study makes it clear that there are no standards for managing psychiatric emergencies.

## Introduction

The number of Emergency Department (ED) visits for psychiatric and substance use disorders has been rising disproportionately. During the COVID-19 pandemic, there has been widespread and increased awareness of the growing prevalence of mental health and substance use disorders. An important contribution to the current conversation is an understanding of the trends prior to the pandemic. For years prior to the pandemic, the rate of psychiatric illness had been increasing. Between 2006 and 2014, the rate of ED visits for mental health disorders increased by 44% while the rate of medical visits increased by 12%. ED visits for mood disorders, anxiety disorders, and schizophrenia and other psychotic disorders increased by 34%, 37%, and 54%, respectively [1]. The rates of ED visits for alcohol and substance-related disorders increased the most (76% and 73%, respectively) with alcohol-related disorders comprising 1.5 million visits in 2014 [1]. Despite the increasing need for mental health care, the number of psychiatric inpatient beds available has been steadily decreasing, further exacerbating a mismatch in the supply and demand of psychiatric services. Beginning in the 1960s, deinstitutionalization leads to a steady decline in the number of psychiatric beds. In 1970 the United States had over 450,000 psychiatric inpatient and residential beds available for a population of 205 million; in 2014 that number dropped to under 180,000 beds for a population of 318 million [2]. While the original intent was to replace psychiatric institutions with outpatient services, this failed to materialize. Adequate community mental health services are not available and 44% of patients experiencing serious mental illness reported perceived unmet needs in 2017 [3]. In 2015 just 10.8% of people over the age of 12 who met the criteria for substance use treatment, received such treatment [4]. For a subset of patients with both psychiatric and substance use diagnoses (co-occurring disorders), barriers to treatment can be even greater [5].

Providing care for patients with mental health disorders (defined as psychiatric and substance use disorders) can be challenging and requires significant and diverse resources due to complex needs. Outpatient resources in the community, housing, medication assistance, and crisis support treatment are highly cited needs of this population [6]. Patients with co-occurring disorders require more specialized care that can be even harder to come by. A core challenge in providing mental health care is inadequate funding. Although the rates of uninsured have been dropping in recent years due to the Affordable Care Act, in 2017 over 28 million people did not have insurance at any point during the year [7]. Moreover, adults with mental illness are uninsured at significantly higher rates than those without mental illness [8]; and for those with coverage, insurance plan “carve-outs” often specifically exclude mental and behavioral health services [9]. Due to this lack of access, mental health patients may put off seeking care and ultimately, when in crisis, go to the ED. Consequently, there has been a widespread surge in mental health ED visits [10].

Mental health patients experience some of the longest LOS in EDs. In a nationwide survey of over 6,000 EDs, more than 70% reported holding psychiatric patients for hours or days - 10% reported “boarding” patients for several weeks when psychiatric beds were not available [11]. However, the literature detailing factors associated with prolonged LOS is relatively sparse with many studies limited to only a few centers and national studies using older data [12]. A deeper dive into mental health ED visits are warranted to identify mutable factors that will inform the development of programs targeting this issue. We investigate ED length of stay (LOS) for mental health compared to non-mental health visits by disposition, diagnosis, and region. Studying visit disposition of admission, transfer, or discharge will provide insight into presumed resource need and availability for mental health vs. medical patients. We will also examine the mental health subpopulations of psychiatric vs. substance use vs. co-occurring disorders. Finally, we will explore the differences in ED use for mental health conditions across the US [10], by comparing regional mental health ED LOS and disposition patterns.

## Materials and methods

Dataset

We used the National Center for Health Statistics’ National Hospital Ambulatory Medical Care Survey (NHAMCS), for years 2009 through 2015. NHAMCS is an annual survey resulting in a national sample of ambulatory care visits within the United States. A three-stage probability sampling design is employed; geographic primary sampling units are defined, hospitals within each primary sampling unit are selected, and finally EDs are sampled [13]. All acute care hospitals not including federal, military, and Veterans Administration hospitals are eligible. Data is obtained by United States Census interviewers.

Study population

Visits to EDs by patients 18-64 years of age were included in the study. Visits were categorized into mental health and medical groupings, using primary ICD-9 diagnostic codes and E-codes. Visits with a disposition of left before triage, left after triage, left against medical advice, died in ED, and died on arrival were excluded. The mental health group included visits with ICD-9 codes 291.0-316.0, excluding 305.1 (tobacco use), and E-codes indicating suicide and self-inflicted injury (E950-E958). The medical group included all other visits. The mental health visit category was then further divided into three mutually exclusive subpopulations: psychiatric, substance use, and co-occurring disorders (both psychiatric and substance use diagnoses). The psychiatric and substance use visits were determined by sole primary ICD-9 psychiatric and substance use diagnostic codes. Co-occurring disorders visits were made by patients who had both psychiatric and substance use ICD-9 codes.

Variables and outcomes

The study population was categorized into five standard age groups: 18-29, 30-39, 40-49, 50-59, and 60-64 years [14]. Sex, which had less than 1% total missing, was imputed by the NHAMCS data center. Because there was substantial missingness for the race and ethnicity variables, we used the data center’s calculated race/ethnicity variable, which collapsed race and ethnicity and imputed missing data. The final race/ethnicity category included Non-Hispanic White, Non-Hispanic Black, Hispanic, and Non-Hispanic Other. Insurance categories were private insurance, Medicare, Medicaid, self-pay, and others. Homeless status was determined from the patient's residence variable. Regions consisted of four geographic areas of the United States as determined by the US Bureau of the Census [15]. We collapsed visit disposition into three groups: discharge, admit, and transfer. The main outcome was LOS which was defined as the time of ED arrival to discharge in hours. Median LOS was calculated, but because of the large variability in LOS, we also examine LOS using benchmarks of six and 12 hours to ensure findings were not due to outliers. More details for NHAMCS variables can be found in the online data documentation [16].

Data analysis

In order to obtain national estimates, strata, weights, and probability sampling unit variables were employed in accordance with NHAMCS data file documentation on ultimate cluster models [16]. All analysis was completed using weighted survey data. Data set-up and analysis were completed using Stata 15 (StataCorp, College Station, TX).

First, we calculated and compared demographic characteristics for the mental health and medical visit groups. A significance level of p<0.005 was determined using the Bonferroni correction for multiple comparisons. Odds ratios, adjusted for age group, sex, race/ethnicity, insurance type, region, and year, were calculated to compare discharge disposition patterns and LOS of the two groups. The median and interquartile range (IQR) of LOS for the mental health and medical groups were calculated by disposition and adjusted odds ratios were used for comparison

Mental Health Subpopulations

Next, we calculated and compared the demographics of the three mental health subpopulations (psychiatric diagnosis, substance use diagnosis, and co-occurring disorders). A significance level of p<0.005 was determined using the Bonferroni correction for multiple comparisons.

The median and IQR of LOS for each group were calculated by disposition. Additional LOS comparisons were made using odds ratios, controlling for age group, sex, race/ethnicity, insurance type, region, and year.

In a secondary analysis, visits for suicide or self-inflicted injury were examined to determine if there were any significant differences between this group and the broader mental health group due to the higher likelihood of concomitant medical problems.

Regional Variation

Finally, we calculated the demographics of the mental health groups for each region (Northeast, Midwest, South, and West). The proportion of visits resulting in discharge, admission, and transfer was compared for mental health vs. medical groups by region. A significance level of p<0.005 was determined using the Bonferroni correction for multiple comparisons.

Next, the median and IQR of LOS was calculated for both mental health and medical groups by region and disposition. Additional comparisons of LOS by region and disposition were made using odds ratios, adjusted for age group, sex, race/ethnicity, insurance type, and year.

## Results

Of an estimated 555 million ED visits over seven years, 5% were mental health and 95% were medical. ED visits for mental health disorders were more likely to be by patients that were male, homeless, and had public insurance (all p<0.005) (Table 1).

**Table 1:** Demographics of 555 million National Hospital Ambulatory Medical Care Survey (NHAMCS) mental health and medical visits (2009-2015) ^a^ weighted visits; ^b^ includes imputed data; SD = standard deviation; IQR = interquartile range (75th percentile-25th percentile)

	Mental Health		Medical	
	N in 1000’s ^a^	Percent	N in 1000’s ^a^	Percent
Total Visits	28,945	5	526,246	95
Age, years				
Mean (SD)	37.9 (14.3)		38.3 (13.2)	
18-29	9,337	32	173,067	33
30-39	6,562	23	114,933	22
40-49	6,566	23	104,693	20
50-59	4,959	17	97,680	19
60-64	1,521	5	35,874	7
Sex				
Female	12,924	45	304,803	58
Male	16,020	55	221,443	42
Race/Ethnicity^b^				
White, Non-Hispanic	18,811	65	311,125	59
Black, Non-Hispanic	5,481	19	127,911	24
Hispanic	3,839	13	72,090	14
Other, Non-Hispanic	813	3	15,120	3
Insurance				
Private	7,128	25	183,472	35
Medicare	3,345	12	46,658	9
Medicaid	8,242	28	126,792	24
Self-pay	5,991	21	96,954	18
Other	4,238	15	72,370	14
Homeless	1,495	5	3,087	1
Region				
Northeast	6,657	23	91,909	17
Midwest	6,984	24	124,603	24
South	8,357	29	204,464	39
West	6,947	24	105,270	20
Disposition				
Admit	4,582	16	46,092	9
Transfer	3,604	12	8,268	2
Discharge	20,759	72	471,887	90
Length of Stay, hours				
Median (IQR)	3.7 (4.7)		2.6 (2.7)	
> 6 hours	9,414	33	79,824	15
> 12 hours	4,183	14	35,630	7

In adjusted results, mental health vs. medical visits were more likely to result in a disposition of admission (adjusted odds ratio (AOR) 2.0, 95% CI 1.8-2.3) or transfer (AOR 8.8, 95% CI 7.6-10.2). Mental health visits were also significantly more likely to have a LOS >6 and >12 hours (Table 2).

**Table 2:** Odds of ED LOS >6 and >12 hours for mental health compared to medical visits overall and by disposition. *adjusted for age group, sex, race/ethnicity, insurance type, region, and year; AOR = adjusted odds ratio; CI = 95% confidence interval; ED LOS = emergency department length of stay

LOS	AOR (CI)*
Overall	
>6 hours	2.7 (2.5-3.0)
>12 hours	2.2 (1.9-2.6)
Admission	
>6 hours	1.1 (0.9-1.3)
>12 hours	1.5 (1.2-1.8)
Transfer	
>6 hours	3.0 (2.3-3.8)
>12 hours	2.7 (2.0-3.7)
Discharge	
>6 hours	2.5 (2.2-2.8)
>12 hours	1.8 (1.5-2.2)

**Figure 1:**
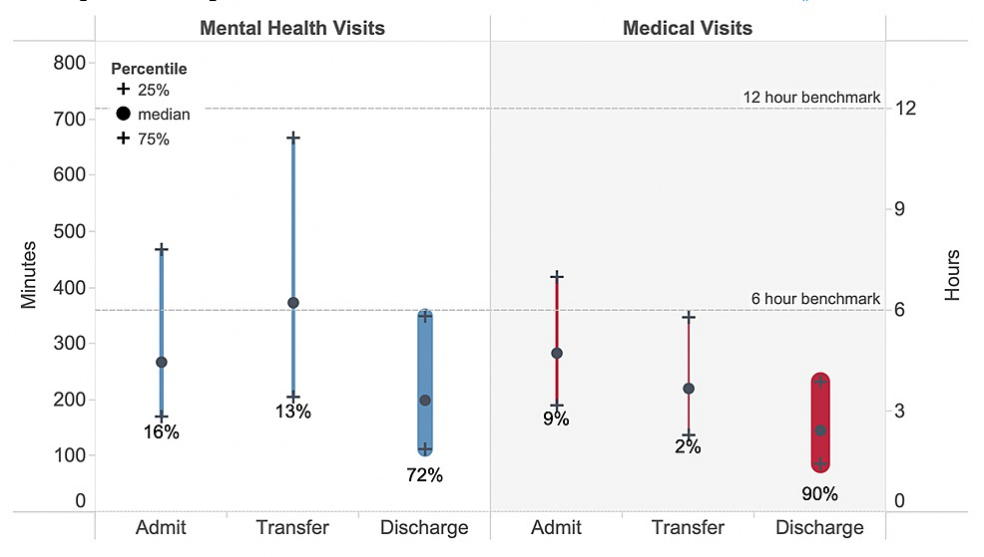
Comparison of mental health and medical visit ED LOS in minutes/hours for dispositions of admission, transfer, and discharge. The width of the bars shows the percent of visits resulting in each disposition. Dotted lines show six- and 12-hour benchmarks. ED LOS = emergency department length of stay

Mental health visits with subsequent transfer had the longest LOS and medical visits resulting in discharge had the shortest LOS (Figure 1).

Mental health vs. medical discharges were more likely to be >6 and >12 hours. Mental health vs. medical admissions were equally likely to last >6 and slightly more likely to last >12 hours. Mental health vs. medical transfers were much more likely to last >6 and >12 hours (Table 2).

Mental health subpopulations

**Figure 2:**
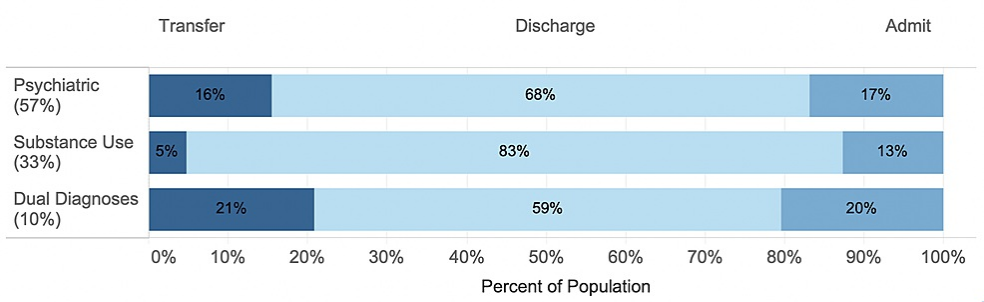
The mental health group by subpopulation and disposition. Each shade of blue represents the proportion of each subpopulation transferred, discharged, or admitted.

Psychiatric diagnoses made up the majority of mental health visits (57%) followed by substance use diagnosis (33%) and co-occurring disorders (10%) (Figure 2).

**Figure 3:**
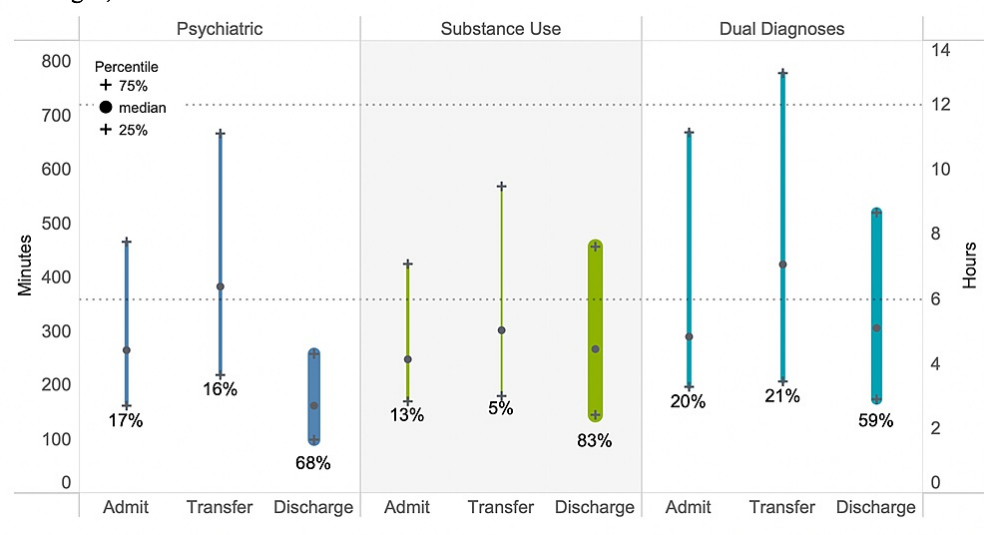
Comparison of psychiatric, substance use, and co-occurring disorders ED LOS in minutes/hours by disposition. The width of each bar represents the proportion of each subpopulation admitted, discharged, or transferred. Dotted lines show six- and 12-hour benchmarks. ED LOS = emergency department length of stay

Within the mental health group, the psychiatric diagnosis visits were by patients that were younger (mean age = 36.9 ± 14.5 years) and more likely to be female (53%) and have Medicare insurance (14%) (all p<0.005). The substance use diagnosis visits were by patients that were older (mean age = 39.6 ± 13.6 years) and were more likely to be male (69%), self-pay (26%), and homeless (8%) (all p<0.005). The co-occurring disorders group had a mean age of 38.1 ± 14.7 years were 60% male, and 7% were homeless. Race/ethnicity was similar for all three groups. More detailed demographics of the three mental health subpopulations are available upon request. Disposition patterns for the three groups were varied (Figure 2). Co-occurring disorders related visits were the most likely to result in admission or transfer and substance use related visits were the most likely to result in discharge (p<0.005). Co-occurring disorders visits had the longest LOS regardless of discharge disposition (Figure 3).

In adjusted analysis, we found that co-occurring disorders visits were significantly more likely to be >6 and >12 hours regardless of disposition (Table 3).

**Table 3:** Odds of ED LOS >6 and >12 hours for psychiatric, substance use, and co-occurring disorders visits compared to medical visits overall and by disposition. *adjusted for age group, sex, race/ethnicity, insurance type, region, and year; AOR = Adjusted odds ratio; CI = 95% confidence interval; ED LOS = emergency department length of stay

LOS	AOR (CI)*		
	Psychiatric	Substance Use	Co-Occurring Disorders
Overall			
>6 hours	2.2 (1.9-2.4)	3.2 (2.8-3.7)	4.8 (3.7-6.2)
>12 hours	2.1 (1.8-2.5)	2.1 (1.7-2.6)	3.5 (2.5-4.8)
Admit			
>6 hours	1.2 (0.9-1.5)	0.9 (0.6-1.2)	1.5 (1.0-2.4)
>12 hours	1.5 (1.1-2.0)	1.2 (0.8-1.8)	2.0 (1.3-3.3)
Transfer			
>6 hours	3.1 (2.4-4.1)	2.0 (1.2-3.5)	3.2 (1.9-5.3)
>12 hours	2.8 (2.0-3.9)	2.0 (1.0-3.9)	3.2 (1.9-5.5)
Discharge			
>6 hours	1.5 (1.2-1.7)	3.9 (3.3-4.6)	4.7 (3.4-6.5)
>12 hours	1.4 (1.1-1.8)	2.3 (1.8-2.9)	2.8 (1.8-4.3)

The odds of substance use visits were especially likely to be prolonged when the resulting disposition was discharge (Table 3). For psychiatric visits, the odds of visits being >6 and >12 hours were highest when the visit resulted in transfer (Table 3).

Of all mental health visits, 11% had an E-code of suicide/self-inflicted injury. Of these suicide/self-inflicted injury visits, 79% were within the psychiatric diagnosis group, 6% were in the substance use group, and 15% were in the co-occurring disorders group. The LOS for the suicide/self-inflicted injury visits was not significantly different from those of mental health visits for other diagnoses (p=0.259).

Regional variation

**Figure 4:**
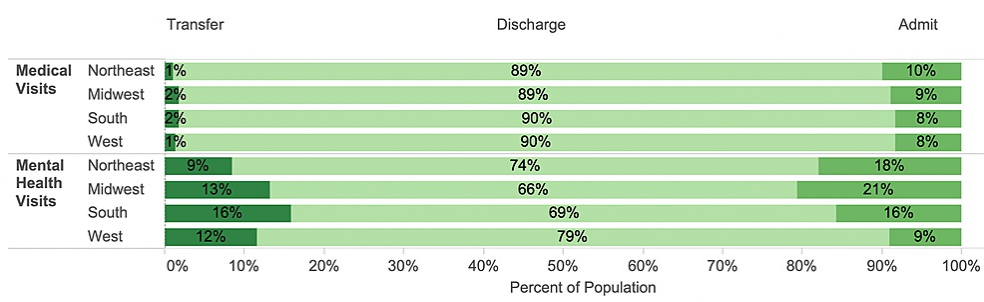
The mental health and medical groups by region and disposition. Each shade of green represents the proportion of mental health and medical visits in each region resulting in transfer, discharge, and admission.

The estimated 29 million mental health visits comprised 7%, 5%, 4%, and 6% of the visits in the Northeast, Midwest, South, and West, respectively. Three regions had mental health visits by mostly male patients (Northeast, 60%; Midwest, 56%; and West, 59%) while the South had visits by mostly female patients (51%). The Northeast had the most Medicaid visits (35%) and the South had the least (22%). The West and Northeast had the most visits by homeless patients (8% and 6%, respectively). More detailed demographics for each of the regions are available on request. Disposition patterns for medical visits were similar across all four regions. Approximately 89%-90% were discharged, 8%-10% were admitted, and 1%-2% were transferred (Figure 1). The breakdown of disposition was more diverse for mental health visits across regions (Figure 4).

The Midwest discharged the least and the West discharged the most (p<0.005). Both the Northeast and Midwest were more likely to admit than transfer (both p<0.005). In the West and South, patients were not significantly more likely to be transferred or admitted (p=0.1, p=1.0, respectively).

**Figure 5:**
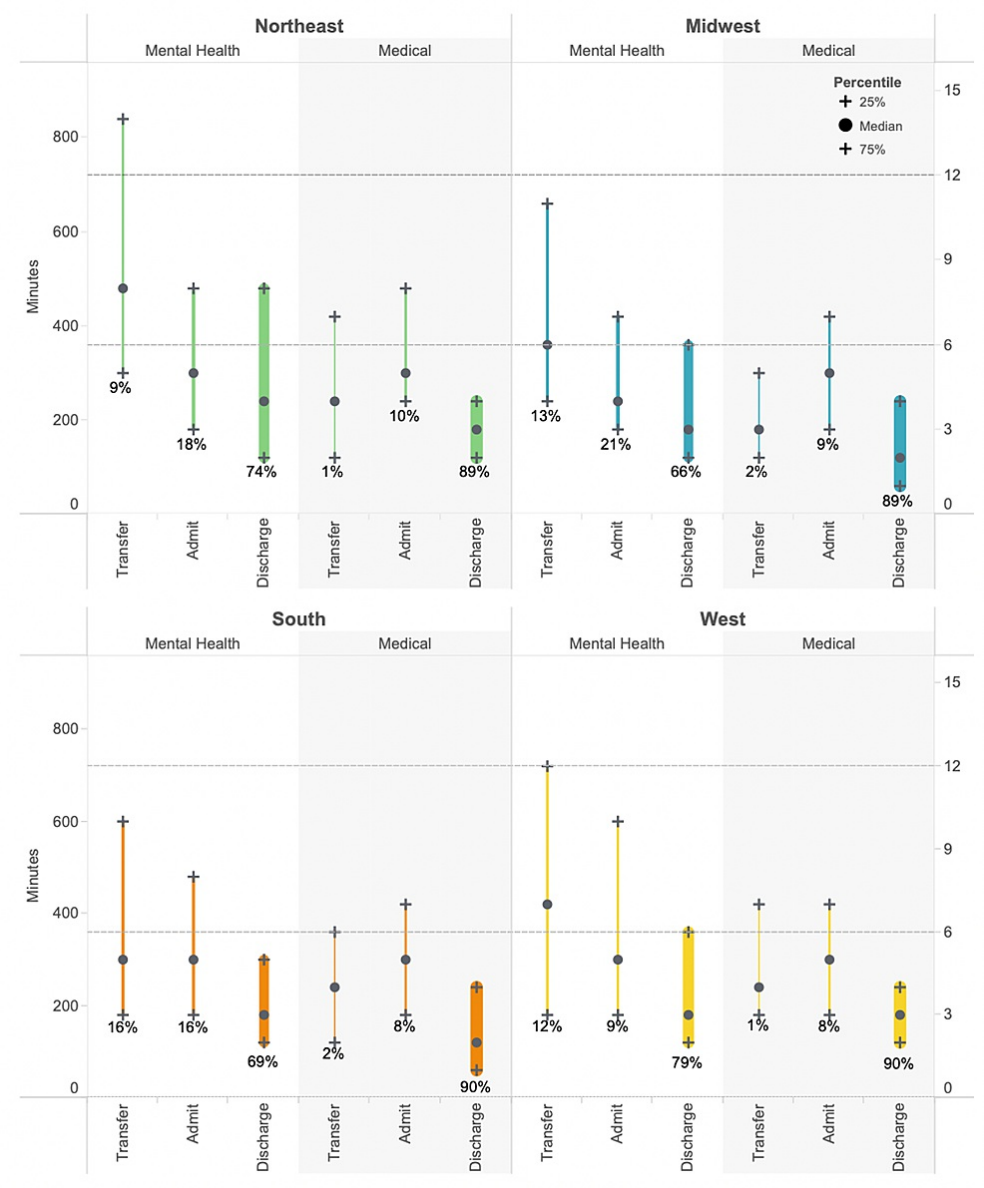
Comparing mental health and medical visit ED LOS in minutes/hours by region and disposition. The width of each bar represents the proportion of each subpopulation admitted, discharged, or transferred. Dotted lines show six- and 12-hour benchmarks. ED LOS = emergency department length of stay

The LOS median (IQR) for mental health visits was 4.6 (5.8), 3.9 (4.4), 3.3 (4.0), and 3.5 (4.4) hours for the Northeast, Midwest, South, and West, respectively. There were differences in the extent of LOS variation for mental health visits for each region and disposition which were not reflected in the medical visits (Figure 5).

In adjusted analysis we found that, compared to medical visits, the Northeast exhibited the highest likelihood of extended ED LOS for mental health visits regardless of disposition; the South was the least likely to have extended LOS for mental health visits (Table 4).

**Table 4:** Odds of ED LOS >6 and >12 hours for the for mental health compared to medical visits for each region overall and by disposition. *adjusted for age group, sex, race/ethnicity, insurance type, and year; AOR = Adjusted odds ratio; CI = 95% confidence interval; ED LOS = emergency department length of stay

LOS	AOR (95% CI)*			
	Northeast	Midwest	South	West
Overall				
>6 hours	4.4 (3.6-5.2)	2.6 (2.1-3.3)	2.2 (1.8-2.6)	2.5 (2.0-3.1)
>12 hours	3.4 (2.8-4.2)	2.0 (1.5-2.8)	2.0 (1.5-2.7)	2.1 (1.6-2.8)
Admission				
>6 hours	1.0 (0.7-1.4)	1.1 (0.8-1.6)	1.2 (0.9-1.7)	1.2 (0.7-1.9)
>12 hours	1.2 (0.8-1.8)	1.5 (0.9-2.3)	1.8 (1.2-2.8)	1.5 (0.9-2.6)
Transfer				
>6 hours	5.2 (3.1-8.7)	3.3 (1.9-5.5)	2.4 (1.6-3.5)	2.7 (1.5-4.8)
>12 hours	3.3 (1.9-5.8)	2.2 (1.2-4.0)	3.0 (1.6-5.5)	2.4 (1.2-4.8)
Discharge				
>6 hours	3.2 (2.6-3.9)	2.9 (2.4-3.6)	2.0 (1.7-2.4)	2.0 (1.6-2.6)
>12 hours	2.2 (1.7-2.8)	2.2 (1.4-3.3)	1.8 (1.3-2.5)	1.4 (1.0-2.1)

## Discussion

We found increased and substantial variation in LOS for mental health compared to medical visits from an analysis of an estimated 555 million US ED visits from 2009 to 2015. The significant variation in LOS persisted when the data were examined by the mental health subpopulation. When examined by region, medical visit disposition patterns were consistent, while those of mental health visits varied markedly. The significant variations seen in the disposition of mental health emergencies indicate there is vast room for improvement to standardize disposition, LOS, and ultimately care models for this vulnerable population.

Our study found that mental health ED visits continued to be substantially longer than medical ED visits [12]. LOS benchmarks have long been used in ED operations as an indicator of the quality of care and patient satisfaction. There are time-to-care benchmarks for many medical conditions with the understanding that longer LOS is associated with worse outcomes. Yet, there are no widely accepted standards for time-to-care for mental health emergencies and it is not unusual for mental health ED visits to last 12 hours or longer. A possible contributor to the increased LOS for transferred and discharged mental health patients is the need for specialist assessment. The traditional emergency mental health care model is ED management with psychiatry consultation. Community and rural centers may have a lack of specialization and in-house mental health professionals may not be available.

In addition to the negative effects of prolonged ED LOS for individual mental health patients, increased LOS can contribute to crowding, which is deleterious for all ED patients. LOS is a well-accepted surrogate of ED crowding and has been associated with adverse effects such as decreased timeliness and quality of care, falling patient satisfaction, and increased rates of medication errors. With ED crowding, there is pressure to discharge patients prematurely and beds that are being taken up by mental health patients cannot be used for others arriving with emergent conditions. Thus, there is a cascade of effects that have implications for the quality of care of all ED patients. The literature on the negative impacts of long LOS on all types of patient care is robust. Decreasing time to treatment is critical for achieving goal outcomes for a multitude of common presenting diagnoses, like stroke [17], acute myocardial infarction [18], and sepsis [19], thus crowding from prolonged mental health LOS impacts the outcomes of medical patients in addition to the obvious adverse impact on mental health patients [20].

In this study, we also explored the role that disposition plays in visiting LOS. Disposition is a surrogate measure of resources required for patient care. For visits resulting in discharge, ED LOS for mental health visits was extended compared to medical visits. Barriers to outpatient follow-up are likely bottlenecks. Connecting patients to community resources and programs can be problematic, especially for underinsured patients or for under-resourced communities. Interestingly, mental health visits resulting in admission had similar odds of extended LOS compared to medical visits. This suggests that if the hospital of the original ED visits has adequate resources for mental health patients requiring higher levels of care, then there is no disparity seen in ED LOS.

The LOS for mental health vs. medical visits resulting in the transfer were significantly more likely to be >6 and >12 hours. This was true regardless of mental health subpopulation or region. Medical clearance requirements have been hypothesized to contribute to extended LOS for ED mental health transfers; however, a Massachusetts study found medical clearance to constitute only a small fraction of ED LOS [21]. Transfers may be required for some patients because no psychiatric bed is available at the hospital during the original ED visit. In this scenario transfer is an indicator of inpatient psychiatric care unavailability or inefficiencies in finding psychiatric beds. This rationale supports a commonly cited source of prolonged LOS for mental health patients: a paucity of psychiatric inpatient beds. Long lengths of time between disposition decision and ED departure were found to be the greatest contributor to extended ED LOS for mental health transfers. Several studies have found that mental health ED visits needing transfer are even longer for uninsured patients than for patients with private or Medicare insurance [21]. This points to the even greater challenges of finding inpatient mental health care for underinsured patients. However, in our study, ED LOS for transfers was greatly prolonged despite adjusting for insurance status, signifying there are other factors contributing to the bottleneck between the decision to transfer and actual transfer. Despite recognition of this problem, however, we have not seen, and should likely not expect, an increase in the number of psychiatric beds as state responses are targeting community-based alternatives [2].

Examining the different subsets of mental health-related visits, we found that psychiatric, substance use, and co-occurring disorders subsets varied in disposition and LOS. Substance use visits resulted in the most discharges and co-occurring disorders patients resulted in the most admissions and transfers. We determined that patients with co-occurring disorders experienced the longest LOS compared to those with psychiatric disorders or substance use disorders alone. These findings are consistent with some literature that found co-occurring disorders to be a predictor for extended ED LOS in adolescents [22]. There is a strong association between substance use disorders and mood and anxiety disorders [23]. A psychiatric condition may predispose someone to a substance use disorder or vice versa. Likewise, factors that are known to enhance the risk for substance use disorders, such as environment, prior trauma, or genetic influence, may increase susceptibility to psychiatric conditions [24]. This relationship may have a synergistic effect when it comes to increasing the time needed for treatment. A patient may need to sober prior to being evaluated for an underlying psychiatric condition or a patient’s medical management for problems such as withdrawal or toxicity may defer psychiatric assessment for an extended period of time. Interestingly, our study found that co-occurring disorders patients were not demographically distinct from the psychiatric and substance use patients, but fell between the two groups in age, sex, race, insurance, and homelessness. However, this group still exhibits increased use of the ED when compared to substance use disorder patients. The increased admission and transfer rates of this population may indicate complexities in care and severity of illness which lead to the extended ED LOS. Inpatient psychiatric facilities may not have the capacity to manage patients with chemical dependence, likewise, additional treatment facilities may be unable to manage coexisting psychiatric illnesses. Further investigation into what factors are influencing their prolonged LOS is warranted.

Not previously documented, our analysis revealed significant regional variation in ED management of mental health patients not mirrored in the medical patients. For example, we found that the Northeast had both the largest percentage of ED visits related to mental health and the longest mental health visit LOS with 20% lasting over 12 hours. However, according to the Substance Abuse and Mental Health Services Administration (SAMHSA), the Northeast had the lowest estimate of serious mental illness between 2012 and 2014 (4%) [25]. Moreover, in 2014, the Northeast was also the only region to have the recommended minimum of 26.5 inpatient beds per 100,000 people in all states [2]. We also found that 34% of mental health visits required admission or transfer in the Midwest but only 21% in the West. It is unlikely that the acuity of mental health emergencies across the nation is so variable that the need for acute mental health care is the only factor at play. Perhaps some of this difference can be attributed to repeat visits. Repeat visits are ubiquitous in EDs and may contribute to a greater proportion of mental health ED visits in the West [26]. Our study found that the West had more mental health visits by homeless patients. Further investigation is needed to determine what mutable factors influence these differences.

One of the mutable factors that have changed significantly since the data were collected in 2015, is the effort to strengthen community resources. As with many healthcare systems, these newly strengthened resources were strained during the overwhelming need for mental health support in the pandemic. A notable change that occurred during the pandemic is the widespread adoption of telehealth, which may become an important tool to support faster access to specialized mental health evaluation in the ED, especially for hospitals that do not have in-house psychiatric specialists. 

Limitations

This study has several limitations to be considered. First, this investigation uses the NHAMCS database, which has multiple known limitations including chart abstraction mistakes, incongruent longitudinal data collection, and variables with significant missingness [27]. Second, the data were from 2009 to 2015 and may not reflect current trends. It does show the context in which the current trends in mental health crisis management developed. Despite these limitations, the dataset offers valuable LOS information not obtainable elsewhere. These data have been used by others to better understand overarching trends in ED utilization [28]. We also recognize that psychiatric diagnoses are seldom made in the ED, thus visits for initial presentation might not capture the entire mental health ED population. To remedy this, we kept sub-diagnoses broad, only differentiating substance use diagnoses vs. psychiatric. Lastly, we were unable to include the most recent NHAMCS data in this study because ED LOS data for 2016 and 2017 were not released due to published data quality issues [16].

## Conclusions

ED visits continue to be longer for mental health vs. medical patients. For mental health visits resulting in transfer and for co-occurring disorders (psychiatric and substance use), ED visits were especially long. Regionally, there is significant variation in mental health ED disposition patterns. This suggests a need for standards in the management of mental health emergencies. Increased resources directed toward mental health patients would decrease crowding and improve care for all ED patients.
